# Personal

**Published:** 1907-10

**Authors:** 


### PERSONAL.

HON. T. GUILFORD SMITH, of Buffalo, regent of the University of the State of New York, tendered a reception to the Hon. Andrew S. Draper, state commissioner of education, at the University club, Tuesday evening, September 17, 1907, immediately after Dr. Draper's address before the State Historical Society at the Buffalo Historical Society's building referred to elsewhere. The reception was attended by representative educators of the city and other prominent citizens and by the out-of-town members of the state historical society.

Dr. James W. Putnam, of Buffalo, one of the associate editors of the *Buffalo Medical Journal,* who sailed for Europe on July 5th, with Mrs. Putnam and their son, Osborne, returned home September 24th, and has resumed his professional practice. The summer was spent in France, Switzerland, Holland and England.

Dr. Putnam attended the International Congress of Neurology and Psychiatry held at Amsterdam, September 2-7, where he read a paper by invitation on "The clinical consideration of paralyses of sudden onset and rapid progress."

---

Dr. F. Park Lewis, of Buffalo, addressed the American Academy of Ophthalmology and Oto-Laryngology at the annual meeting held at Louisville, September 26-29, 1907, on the subject of ophthalmia neonatorum with reference to its prophylaxis. He will present the same subject at the thirty-fifth annual meeting of the American Public Health Association to be held at Atlantic City, September 30 to October 4, 1907.

---

Dr. William C. Krauss, of Buffalo, assistant editor of the *Buffalo Medical Journal,* who has been spending the summer in Europe, returned home October 2, and resumed his medical practice. He spent considerable time in Munich, attending the Mozart and Wagner festivals and enjoying other musical treats.

---

Dr. John H. Grant, of Buffalo, was elected surgeon general of the Spanish War Veterans' Association at the recent meeting held at Sandusky, O.

---

Dr. G. Lane Taneyhill, of Baltimore, was elected surgeon-general of the Grand Army of the Republic at its forty-first annual meeting recently held at Saratoga Springs.

---

Dr. William Gaertner, of Buffalo, was elected president of the Buffalo Orpheus Society at its annual meeting held early in September.

---

Dr. Francis A. Drake, of Buffalo, was elected High Physician of the Independent Order of Foresters, high court of the State of New York, at its recent annual meeting held at Jamestown, N. Y.

---

Dr. Howard W. Longyear, of Detroit, recently made a hurried trip to Europe on account of the illness of his son-in-law, who is in Switzerland. Professor Kocher, of Berne, operated on Mr. McGraw for bronchocele, soon after Dr. Longyear's arrival from which he has made a safe recovery. Dr. Longyear returned in season to attend the annual meeting of the American Association of Obstetricians and Gynecologists at Detroit.

---

Dr. Joseph Phineas Runyan, of Little Rock, Ark., was re-elected dean of the College of Physicians and Surgeons, Little Rock, at the meeting of the faculty held September 2, 1907.

---

Dr. James F. W. Ross, of Toronto, was chosen as the first president of the recently created Academy of Medicine of the Canadian metropolis. The eminence Dr. Ross has attained as one of the most foremost abdominal surgeons in North America, entitles him to this distinction paid by his colleagues in his native city.

---

Dr. Benjamin H. Grove, of Buffalo, who, with Mrs. Grove, has been spending the summer in European travel, has returned home and resumed his ophthalmic practice.

